# *Aeromonas* spp. as possible bacterial indicator for monitoring antibiotic resistance in seafood

**DOI:** 10.3389/fmicb.2025.1721645

**Published:** 2026-01-20

**Authors:** Elisa Albini, Biagio Caponi, Silvia Pieralisi, Serenella Orsini, Francesca Blasi, Luisa Massaccesi, Carmen Maresca, Eleonora Scoccia, Alessandro Fiorucci, Valeria Michelacci, Paola Chiani, Manuela Marra, Maria Carollo, Francesca Romana Massacci, Giovanni Pezzotti, Francesca Leoni, Chiara Francesca Magistrali

**Affiliations:** 1 Istituto Zooprofilattico Sperimentale Dell’Umbria e Delle Marche “Togo Rosati”, Perugia, Italy; 2 National Research Council of Italy, Institute for Agriculture and Forestry System in the Mediterranean (ISAFOM-CNR), Perugia, Italy; 3 Dipartimento di Sicurezza Alimentare, Nutrizione e Sanità Pubblica Veterinaria, Istituto Superiore di Sanità, Roma, Italy; 4 Servizio Grandi Strumentazioni e Core Facilities, Istituto Superiore di Sanità, Roma, Italy; 5 Istituto Zooprofilattico Sperimentale Della Lombardia e Dell’Emilia Romagna “Bruno Ubertini”, Brescia, Italy

**Keywords:** *Aeromonas*, antibiotic-resistance, epidemiologic cut-off, seafood, bacterial indicator

## Abstract

Fishery and aquaculture products increasingly represent an important food source for the world population. The intensification of aquaculture guided an increase in the use of antimicrobials in this sector as well. The aquatic environment represents a link among humans, animals and environmental antibiotic resistance. The rising problem of antibiotic resistance leads to the need for the identification of indicator bacteria and the development of monitoring methods, which are poorly standardized for the aquatic environment. *Aeromonas* spp., a ubiquitous bacterium in aquatic environments, is among the possible candidates as bacterial indicator. The goals of this study were to: (i) evaluate the antibiotic-resistance profile of different antimicrobial classes in *Aeromonas* spp. collected from seafood sold in superstores of central Italy; (ii) propose possible epidemiological cut-offs for the genus *Aeromonas* and evaluate its effectiveness as a possible bacterial indicator for monitoring antibiotic resistance in seafood. The results obtained revealed a high presence of *Aeromonas* spp. in seafood categories, highlighting its candidature as a bacterial indicator. The presence of isolates resistant to some of the tested antibiotics has been described, even if in rare occasion. This data raises concerns about the food safety of seafood for consumption, which may represent a risk for public health and consumers. In this study, we described the epidemiological cut-offs which agree with the literature, representing a contribution to the definition of reliable epidemiological cut-offs for the genus *Aeromonas* spp.

## Introduction

In the livestock sector, the introduction of antimicrobials has allowed a more intensive and industrial development. Fish farms and aquaculture contribute to producing 52% of the total fish consumed in the world (FAO, 2020). However, the intensification of fish farming has led to an increase in the use of antimicrobials in this sector too. The use of antibiotics to prevent the emergence and rapid spread of bacterial infections is a widespread practice (Okocha et al., 2018). In most European countries, antibiotics authorized for use in aquaculture are oxytetracycline, florfenicol, sarafloxacin, erythromycin, and sulfonamides (trimethoprim-enhanced) (Kümmerer, 2009), while in the United States, oxytetracycline, florfenicol, and sulfadimethoxine are authorized (FDA, 2025).

The massive and/or irrational use of antimicrobials in aquaculture poses a risk problem for food safety, for the health of humans, animals and ecosystems (Miller and Harbottle, 2018), causing the spread of antimicrobial resistance (AMR) in the aquatic environment. Resistant bacteria, resistance genes, and residues of antibiotics used in aquaculture may spread through fish products and enter the food chain (Okocha et al., 2018; Lee et al., 2019). A number of studies have found that many aquatic bacteria harbour a large variety of mobile genetic elements such as plasmids, integrons, and transposons that can easily move, recombine and mobilise, favouring the emergence of new mobile combinations of antimicrobial resistance genes (ARGs), giving the bacteria the ability to rapidly adapt to new environments where antimicrobials are present (Sørum, 2008; Cabello et al., 2013).

In this scenario, the need for surveillance of AMR in seafood has become evident, as highlighted by several authors (Grevskott et al., 2017; Banerjee and Farber, 2018; Schar et al., 2021; Albini et al., 2024). Continuous monitoring is essential to track the dissemination of resistant bacteria in the marine environment, as well as to guide regulatory measures for the global fish trade.

In terrestrial mammals, surveillance of AMR is based on testing commensal bacterial, with *Escherichia coli* used as an indicator of AMR (Usui et al., 2016; EFSA, 2024). However, *E. coli* is not a common inhabitant of aquatic environments. On the contrary, *Aeromonas* spp. is considered ubiquitous in most aquatic environments (Rhodes et al., 2000) and easily isolated from most fish products (Gonçalves Pessoa et al., 2019), and thus it can be a possible candidate as an indicator microorganism.

*Aeromonas* are gram-negative bacilli ubiquitous in aquatic environments (Martin-Carnahan and Joseph, 2005). The taxonomy of this genus, continuously updated, included 36 species (Fernández-Bravo and Figueras, 2020). *Aeromonas* spp. are native to aquatic environments and have been isolated to date from surface, groundwater, drinking, bottled, residual, seawater, and irrigation water (Fernández-Bravo and Figueras, 2020).

Although *Aeromonas* are mainly fish pathogens, several species of *Aeromonas* spp. are considered emerging pathogens as they are responsible for several diseases in humans, mainly gastroenteritis, wound infections and bacteremia/septicemia, causing infections primarily in immunocompromised subjects (Figueras et al., 2005; Janda and Abbott, 2010). Studies have so far reported that the majority of isolates associated with clinical cases can be identified as four species*, A. caviae*, *A. dhakensis*, *A. veronii*, and *A. hydrophila*, with prevalence varying according to the area and the study of origin (Fernández-Bravo and Figueras, 2020; Pessoa et al., 2022). Other species typically associated with fish diseases, such as *A. salmonicida*, have also been reported in human infections (Vincent et al., 2019).

*Aeromonas* spp. are intrinsically resistant to ampicillin, amoxicillin-clavulanate and cefazolin (CLSI, 2018). Additionally, *Aeromonas* spp. isolates have been shown to carry multiple ARGs, as well as mobile genetic elements (MGEs), such as plasmids or transposons, indicating their potential to transfer ARGs to other bacterial species (Piotrowska et al., 2017).

Although antibiotic resistance in clinical isolates of *Aeromonas* spp. has been partially explored, considerably less information is available regarding resistance patterns in strains originating from food or environmental sources.

“Clinical breakpoints” are used to interpret an *in vitro* value of the minimum inhibitory concentration (MIC) and to estimate the odds of therapeutic success to treat infection; “Epidemiological cut-off” values (ECOFFs or ECVs, for EUCAST or CLSI, respectively) allow to recognize any emerging resistance mechanism in bacterial populations studied and categorizing the microorganism in wild type or non-wild type (CLSI, 2025; EUCAST, 2025).

Clinical and Laboratory Standards Institute (CLSI) only provides antimicrobial breakpoints and susceptibility test protocols for *A. salmonicida* (CLSI, 2020a). Recently, the CLSI guideline (VET 04) updated the epidemiological cut-off values (ECVs) for *A. salmonicida* to seven drugs including gentamicin, erythromycin, florfenicol, ormetoprim–sulfadimethoxine, trimethoprim–sulfamethoxazole, oxytetracycline, and oxolinic acid. It also provides the epidemiological cut-off values for *A. hydrophila* to six drugs including gentamicin, erythromycin, florfenicol, oxytetracycline, enrofloxacin, and oxolinic acid. The only MIC or zone diameter breakpoints established by EUCAST^1^ are the clinical breakpoints for *Aeromonas* spp. to six drugs including cefepime, ceftazidime, aztreonam, ciprofloxacin, levofloxacin, and trimethoprim–sulfamethoxazole. However, there is still a paucity of clinical breakpoints and epidemiological cut-off value for the *Aeromonas* genus, likely because most antimicrobial classes are not commonly used in aquaculture. According to the literature, only few studies determining tentative epidemiological cut-off values based on MIC are available (Baron et al., 2017; Lin et al., 2022; Woo et al., 2022). This limitation considerably reduces the use of *Aeromonas* as an indicator of AMR in seafood.

EFSA’s scientific report on technical specifications for an EU-wide baseline survey on antimicrobial resistance in bacteria from aquaculture animals recommended a survey aiming at estimating the occurrence of AMR in *Aeromonas* spp. isolated from Atlantic Salmon, European seabass and trout intended to consumption, at harvesting (at farm/slaughter) (Aerts et al., 2024). Due to the EFSA’s recommendation and since limited data are available in the literature, a more in-depth evaluation is needed to support the hypothesis that *Aeromonas* spp. can be considered a reliable indicator in fish products. The aim of our study was to investigate the prevalence of *Aeromonas* spp. in seafood at retail, to evaluate the ease of detection of *Aeromonas* spp. in different seafood products, both fresh and frozen. In addition, a subset of isolates from various fish products was tested for antimicrobial susceptibility against several clinically important antimicrobial classes to propose possible epidemiological cut-offs values, following internationally accepted methods (Kronvall, 2010). Our final goal was to support the inclusion of *Aeromonas* spp. as an indicator for AMR surveillance in seafood.

## Materials and methods

### Sample preparation and bacteria isolation

Seven categories of samples of raw commercial seafood were chosen basing upon the data about seafood consumption in Italy (Table 1). The number of samples to collect was calculated with a 20% expected prevalence, 95% confidence levels and 10% precision, resulting in 62 samples for each category, for a total of 434 samples. The samples were collected from 10 different hypermarkets in Central Italy between June 2020 and May 2021. A total of 422 out of 434 samples were collected due to the availability of commercial seafood. Information about the origin of the sample [Food and Agriculture Organization (FAO) FAO fishing areas], raising claims (farm-raised *vs*. wild-caught) and sold form (fresh *vs*. frozen) were recorded for all samples. Samples were packed in sterile bags, placed on ice in a cool box, and immediately transported to the laboratory, where they were processed within 2 h after arrival. Frozen fillets were allowed to thaw at an ambient temperature in their original envelopes. The samples were processed following the UNI EN ISO 6887-3, using sterile material. Briefly, portions of fish, fillets, and crustaceans (approx. 25 g each) were cut with sterile scalpels under aseptic conditions. For the bivalves, the 25 grams included flesh and intravalvular liquid. Samples were placed in Stomacher bags, diluted 1:10 with Alkaline Peptone Water, homogenized with Stomacher® and incubated at 30 ± 1 °C per 24–48 h. Then, 10 μL of the suspension was spread on Glutamate Starch Phenol Red Agar (GSP) and the plates were incubated at 22 ± 1 °C per 48 h (Aerts et al., 2024). Yellow colonies on GSP were considered as presumptive *Aeromonas* and they were isolated on Tryptic Soy Agar and incubated at 37 °C for 24 h and stored in 20% glycerol Luria-Bertani medium at −20 °C for further analysis.

**Table 1:** The prevalence rate of *Aeromonas* spp. among seafood category samples.

Seafood category samples	Negative samples		Positive samples		Total
	*N*	%	*N*	%	
A (bass)	1	1.5%	64	94.5%	65
B (anchovy)	9	23.7%	29	76.3%	38
C (cephalopods)	26	38.2%	42	61.7%	68
D (cod)	42	63.6%	24	36.4%	66
E (salmon)	30	50%	30	50%	60
F (bivalves)	18	29.5%	43	70.5%	61
G (crustaceans)	42	65.6%	22	34.4%	64
Total	168	39.8%	254	60.2%	422

### Bacterial isolates identification

The identification of presumptive *Aeromonas* isolates was confirmed at genus level using a MALDI-TOF MS instrument (Bruker Daltonics, Bremen, Germany) with Microflex LT Smart Biotyper with FlexControl Biotyper 3.4 software (Bruker Daltonics, Bremen, Germany) and identification at the species level was based on the partial *gyrB* gene sequencing (Yáñez et al., 2003). Genomic DNA was extracted from 1 mL of overnight cultures grown in Tryptone Soy Broth at 37 °C using the protocol for Gram-negative bacteria of the QIAamp® DNA Mini Kit (Qiagen GmbH, Hilden, Germany), according to manufacturer’s instructions. For PCR amplification, the primers gyrB3-F (5′-TCCGGCGGTCTGCACGGCGT-3′) and gyrB14-R (5′-TTGTCCGGGTTGTACTCGTC-3′) were used to amplify an approximately 1,100 bp *gyrB* gene (Yáñez et al., 2003). All PCR reactions were performed with 50 μL containing 10 μL of 5X Buffer Mix (Promega Corporation, Wisconsin, USA), 1.5 mM MgCl_2_, 0.2 mM of each nucleotide, 0.4 μM each primer, 0.5 U Taq DNA polymerase (Promega Corporation, Wisconsin, USA) and 50–100 ng DNA template. PCR amplification was as follows: initial denaturation at 95 °C for 15 min, 35 cycles of denaturation at 95 °C for 30 s, annealing at 52 °C for 30 s, and extension at 72 °C for 60 s, followed by a final extension at 72 °C for 7 min. PCR products were visualized by electrophoresis in a 1.5% agarose gel in 1 × TAE buffer. PCR products were purified with the High Pure PCR Product Purification Kit (Roche Diagnostics GmbH, Germany), following the manufacturer’s instructions. DNA sequencing was performed using BrilliantDye® Terminator v3.1 Kit (NimaGen BV, The Netherlands), according to manufacturer’s instructions, and using the 3500 Genetic Analyzer (Applied Biosystems, Massachusetts, USA). The sequences were compared with available sequences (>97% nucleotide BLAST similarity) in the GenBank database in the National Center for Biotechnology Information (NCBI).

### Antimicrobial susceptibility testing and determination of MICs values

To estimate the prevalence of the antibiotic resistance of *Aeromonas* spp. isolates, a subset (*n* = 100) was carried out using the Epitools website.^2^ The choice of isolates was based on a blocked randomization, in seven strata each representing a category of seafood (Petrie and Watson, 2013). The selection considered a total population of 422 samples, an expected prevalence of 50%, a confidence level of 95% and a precision of 10%, resulting in 97 isolates, that has been increased to 100 isolates. The information on the 100 *Aeromonas* spp. included in the study are reported in Supplementary Table S1. The *Aeromonas* isolates were tested to determine the MICs of 8 antimicrobial agents using the broth micro-dilution method according to CLSI guidelines (CLSI VET03, 2020). The antimicrobials tested were: nalidixic acid (NAL), cefotaxime (CEF), colistin (COL), erythromycin (E), florfenicol (FFC), gentamicin (GEN), trimethoprim-sulfamethoxazole (SXT) and tetracycline (TE). Since *Aeromonas* are naturally resistance to penicillins, this class of antimicrobial was not tested (Bakken et al., 1988; Batra et al., 2016). A stock solution of each antimicrobial agent at 5120 μg/mL concentration was prepared with the solvent recommended by CLSI (2020a) and aliquots were stored at −20 °C. Antimicrobial susceptibility tests were performed in 96-well microplates (LP ITALIANA, Milano, Italy), testing 12 two-fold dilutions for each antimicrobial (0.016–32 μg/mL). If the MIC values were higher or lower than the highest or lowest concentration measured further dilutions were tested. The inocula were prepared by the colony suspension in cation adjusted Muller−Hinton broth (CAMHB), as recommended (CLSI, 2020a). The plates were incubated at 28 ± 1 °C per 48 h, according to the protocols provided in the CLSI VET03 guideline (CLSI, 2020a). Reference strains *Escherichia coli* ATCC 25922 and *Aeromonas salmonicida* subsp. *salmonicida* ATCC 33658 were used as controls and included in each plate, as recommended by CLSI for this method (CLSI, 2020a, 2020b).

### Epidemiological cut-off values (COWT) determination

Epidemiological cut-off (COwt) values were statistically determined according to the methods proposed by Kronvall (2010). Fully automated and freely available Excel spreadsheet calculators to apply the normalized resistance interpretation (NRI) method (Kronvall, 2010) [available at http://www.bioscand.se/nri/ used with permission from the patent holder, Bioscand AB, TÄBY, Sweden (European patent No 1383913, US Patent No. 7,465,559)] and ECOFFinder MS (available at https://clsi.org/resources/ecoffinder/) were used. Calculations were performed for each antimicrobial to categorize the *Aeromonas* isolates in wild-type (WT) and non-wild-type (NWT), when those placed below or above the COwt, respectively.

### Calculation of MIC50 and MIC90

MIC50 is the MIC required to inhibit 50% bacterial growth, and MIC90 is the MIC required to inhibit 90% bacterial growth. The strains with MIC values greater than the breakpoint value of the 95% or 97.5% confidence interval are all NWT strains. The strains with MIC values less than or equal to the COwt value of the 95% or 97.5% confidence interval are WT strains.

### Genomic analysis

The *Aeromonas* spp. isolates classified as NWT to at least one tested antibiotic were whole genome sequenced. DNA was quantified with the Qubit fluorometer (QubitTM DNA HS Assay, Thermo Fisher Scientific Inc.), the libraries were prepared from 100 ng of DNA using the NEBNext Fast DNA Fragmentation & Library Prep Set for Ion Torrent kit (New England BioLabs, MA, USA) and then sequenced using the Ion Chef and Ion Torrent S5 instruments (Thermo Fisher Scientific, MA, USA) using a 400 bp sequencing protocol and the Ion 510™ & Ion 520™ & Ion 530 kit (Thermo Fisher Scientific, MA, USA). Basic analyses were performed on ARIES web platform for bioinformatic analysis^3^: trimming of the reads was performed by using the tool FASTQ Positional and quality trimming; SPAdes (3.14.1) (Bankevich et al., 2012) and Filter SPAdes repeats tools were used for assembling the reads in contigs for with default parameters; Quast tool (Gurevich et al., 2013) was used to inspect the quality of the contigs and species identification was performed by Ribosomal Multilocus Sequence Typing (rMLST).^4^ The presence in the genomes of antibiotic-resistance genes was analyzed using ResFinder v4.7.2, considering only genes with a ≥95% coverage and ≥90% identity. The raw sequencing data has been submitted to EMBL-ENA (project Accession numbers: PRJEB96839).

### Statistical analysis

Prevalence data were summarized as percentages with corresponding 95% confidence intervals (CI). The chi-square test, using the Pearson’s chi-square, was applied to assess differences in the frequency of positive samples between fresh *vs* frozen and farm-raised *vs* wild-caught samples. A *p-*value < 0.05 was considered statistically significant. All analyses were performed in R version 4.5.0 (Team Core, 2025).

## Results

### Isolation, identification and distribution of isolates

From the starting collection of 422 samples, a total of 254 (60.19%, 95% CI: 55.45%–64.75%) resulted positive for *Aeromonas* spp. (Table 1).

In the seafood category sample A (sea bass) all samples resulted positive except for one, moreover in categories B (anchovy), C (cephalopods), and F (bivalves) more than 60% of the samples were positive (Table 1).

The chi-square test confirmed the presence of a difference between the number of samples positive for *Aeromonas* spp. in fresh and frozen samples. The difference was detected both by considering the total samples and by individually considering the categories in which both the fresh and frozen samples were taken (categories C, D, E and G; *χ*^2^ Pearson: *p* < 0.001), while no association was found for farm-raised *vs* wild-caught seafood (Table 2).

**Table 2:** The prevalence of positive sample and the association between the presence of *Aeromonas* spp. and the sold form (fresh vs. frozen).

Seafood category samples	Fresh			%	95% CI	Frozen			%	95% CI
	Positive samples	Negative samples	Total			Positive samples	Negative samples	Total		
A (bass)	64	1	65	98.46	90.6–99.91	0	0	0	0	-
B (anchovy)	29	9	38	74.36	57.52–86.4	0	0	0	0	-
C (cephalopods)	37	2	39	94.87	81.37–99.11	5	24	29	17.24	6.53–36.58
D (cod)	12	2	14	85.71	56.15–97.49	12	40	52	23.07	12.98–37.17
E (salmon)	18	5	23	78.26	55.79–91.71	12	25	37	32.43	18.55–49.89
F (bivalves)	43	18	61	70.49	57.26–81.13	0	0	0	0	-
G (crustaceans)	10	2	12	83.33	50.88–97.06	12	40	52	23.08	12.98–37.17
Total	213	39	252	84.52	79.33–88.64	41	129	170	24.12	18.04–31.39

The percentage refers to positive samples for each seafood category samples.

The prevalence of the antibiotic resistance of *Aeromonas* spp. isolates was tested on randomly selected subset (Supplementary Table S1). All isolates were subjected to *gyrB* gene sequencing for species identification (Table 3). Among the 10 bacterial species identified, the most prevalent was *A. salmonicida* (47%), followed by *A. media* (21%). The other species were represented by a few or only one isolate (Table 3). *A. salmonicida* and *A. media* were isolated across all seafood categories, whereas *A. rivipollensis* was not recovered in A and B seafood categories samples. The two isolates of *A. hydrophila* and *A. allosaccharophila* were isolated from A and B and A and F seafood categories samples, respectively. The only species belonging to *A. caviae* was isolated from D seafood category sample (Supplementary Table S2).

**Table 3:** Species identification of *Aeromonas* spp. isolates.

Bacteria species	Total
*Aeromonas allosaccharophila*	2
*Aeromonas bivalvium*	7
*Aeromonas caviae*	1
*Aeromonas crassostreae*	3
*Aeromonas hydrophila*	2
*Aeromonas media*	21
*Aeromonas molluscorum*	4
*Aeromonas rivipollensis*	7
*Aeromonas salmonicida*	47
*Aeromonas veronii*	6
Total	100

### Antimicrobials susceptibility and establishment of epidemiological cut-off values

We evaluated the distributions of the MIC values for each antibiotic and the corresponding MIC_50_ and MIC_90_ (Table 4). For each MIC determination assay, the results obtained for the reference strains (*Escherichia coli* ATCC 25922 and *Aeromonas salmonicida* subsp. *salmonicida* ATCC 33568) complied with CLSI recommendations (CLSI VET04, 2020). The MICs of three antibiotics (nalidixic acid, trimethoprim-sulfamethoxazole and cefotaxime) exceeded the dilution range and were further tested. Antibiotics with highest MIC values (256 μg/mL) were nalidixic acid (*n* = 3) and trimethoprim-sulfamethoxazole (*n* = 1), while cefotaxime has MIC value lower than the lowest concentration determined (≤0.016, *n* = 1). The other antibiotics tested had MICs that were always within the drug dilution range.

**Table 4:** Distribution of MIC values, MIC_50_ and MIC_90_ (μg/ml) in 100 *Aeromonas* spp. isolates and interpretative criteria of the epidemiological cut-offs (ECOFF).

Antimicrobial agents^a^	MIC (μg/ml)																COwt	MIC_50_	MIC_90_
	≤0.016	0.016	0.031	0.062	0.125	0.25	0.5	1	2	4	8	16	32	64	128	256			
NAL					35	52	5	1	2			1			1	3	0.5	0.25	0.5
CEF	1	2	6	31	25	25	7	2	1								0.5	0.125	0.25
COL					5	32	54	7	1				1				2	0.5	0.5
E						1			5	15	42	28	9				64	8	16
FFC						1	6	41	41	11							4	2	4
GEN							5	36	46	9	4						8	2	4
SXT						3	40	52	2				1		1	1	4	1	1
TE				1	37	55	2				1	3	1				0.5	0.25	0.25

a Nalidixic acid (NAL), cefotaxime (CEF), colistin (COL), erythromycin (E), florfenicol (FFC), gentamicin (GEN), sulfamethoxazole-trimethoprim (SXT), tetracycline (TE).

The blue lines represent the epidemiological cut-offs (COwt), calculated according to the Kronvall method, 2010. The percentages correspond to the number of isolates tested.

The interpretation of COwt for each antibiotic was calculated following the method of Kronvall (2010). For each antibiotic tested the calculated COwt was as follows: nalidixic acid (NAL) COwt = 0.5, cefotaxime (CEF) COwt = 0.5, colistin (COL) COwt = 2, erythromycin (E) COwt = 64, florfenicol (FFC) COwt = 4, gentamicin (GEN) COwt = 8, sulfamethoxazole + trimethoprim (SXT) COwt = 4, tetracycline (TE) COwt = 0.5. The calculation of the Normalized Resistance Interpretation based on the MIC distribution for each tested antibiotic is described in Supplementary Figure S1.

The MIC distributions for all drugs tested were unimodal. Cefotaxime had the largest COwt value, while the nalidixic acid had the narrowest COwt value (Supplementary Figure S1). For colistin, sulfamethoxazole-trimethoprim and tetracycline, the MIC_50_ and MIC_90_ values were the same: 0.5 μg/mL, 1 μg/mL and 0.25 μg/mL, respectively. For the other antimicrobials tested (nalidixic acid, cefotaxime, erythromycin, florfenicol and gentamicin) differences between MIC_50_ and MIC_90_ were only one dilution. The classification into WT and NWT of the isolates tested for each antibiotic was performed based on the Cowt calculated following the method of Kronvall (2010) (Table 5). We described *n* = 8 NWT isolates for nalidixic acid, *n* = 3 for cefotaxime, *n* = 1 NWT isolate for colistin, *n* = 3 for sulfamethoxazole-trimethoprim and *n* = 5 for tetracycline. All isolates were WT for erythromycin, florfenicol and gentamicin. For individual species, *A. allosaccharophila*, *A. caviae*, *A. crassostreae A. molluscorum* and *A. veronii* resulted WT for all antibiotics tested (Table 5).

**Table 5:** Classification of *Aeromonas* spp. isolates into wild-type (WT) and non-wild-type (NWT) based on the calculated MIC values and epidemiological cut-offs (COwt).

Bacterial species	Total of isolates	NAL		CEF		COL		E		FFC		GEN		SXT		TE	
		NWT (%)	WT (%)	NWT (%)	WT (%)	NWT (%)	WT (%)	NWT (%)	WT (%)	NWT (%)	WT (%)	NWT (%)	WT (%)	NWT (%)	WT (%)	NWT (%)	WT (%)
*A. allosaccharophila*	2	0 (0)	2 (100)	0 (0)	2 (100)	0 (0)	2 (100)	0 (0)	2 (100)	0 (0)	2 (100)	0 (0)	2 (100)	0 (0)	2 (100)	0 (0)	2 (100)
*A. bivalvium*	7	1 (14.3)	6 (85.7)	0 (0)	7 (100)	0 (0)	7 (100)	0 (0)	7 (100)	0 (0)	7 (100)	0 (0)	7 (100)	1 (14.3)	6 (85.7)	0 (0)	7 (100)
*A. caviae*	1	0 (0)	1 (100)	0 (0)	1 (100)	0 (0)	1 (100)	0 (0)	1 (100)	0 (0)	1 (100)	0 (0)	1 (100)	0 (0)	1 (100)	0 (0)	1 (100)
*A. crassostreae*	3	0 (0)	3 (100)	0 (0)	3 (100)	0 (0)	3 (100)	0 (0)	3 (100)	0 (0)	3 (100)	0 (0)	3 (100)	0 (0)	3 (100)	0 (0)	3 (100)
*A. hydrophila*	2	0 (0)	2 (100)	0 (0)	2 (100)	1 (50)	1 (50)	0 (0)	2 (100)	0 (0)	2 (100)	0 (0)	2 (100)	0 (0)	2 (100)	0 (0)	2 (100)
*A. media*	21	3 (14.3)	18 (85.7)	2 (9.5)	19 (90.5)	0 (0)	21 (100)	0 (0)	21 (100)	0 (0)	21 (100)	0 (0)	21 (100)	0 (0)	21 (100)	1 (4.8)	20 (95.2)
*A. molluscorum*	4	0 (0)	4 (100)	0 (0)	4 (100)	0 (0)	4 (100)	0 (0)	4 (100)	0 (0)	4 (100)	0 (0)	4 (100)	0 (0)	4 (100)	0 (0)	4 (100)
*A. rivipollensis*	7	1 (14.3)	6 (85.7)	0 (0)	7 (100)	0 (0)	7 (10)	0 (0)	7 (100)	0 (0)	7 (100)	0 (0)	7 (100)	2 (28.6)	5 (71.4)	3 (42.9)	4 (52.1)
*A. salmonicida*	47	3 (6.4)	44 (93.6)	1 (2.1)	46 (97.9)	0 (0)	47 (100)	0 (0)	47 (100)	0 (0)	47 (100)	0 (0)	47 (100)	0 (0)	47 (100)	1 (2.1)	46 (97.9)
*A. veronii*	6	0 (0)	6 (100)	0 (0)	6 (100)	0 (0)	6 (100)	0 (0)	6 (100)	0 (0)	6 (100)	0 (0)	6 (100)	0 (0)	6 (100)	0 (0)	6 (100)
*Total*	100	8	92	3	97	1	99	0	100	0	100	0	100	3	97	5	95

Nalidixic acid (NAL), cefotaxime (CEF), colistin (COL), erythromycin (E), florfenicol (FFC), gentamicin (GEN), sulfamethoxazole-trimethoprim (SXT), tetracycline (TE).

### Genomic analysis

The *Aeromonas* spp. isolates classified as NWT (*n* = 18) were further investigated by whole genome sequencing. The median number of assembled contigs was 92.5, with a median N50 of 86,958 bp and a median GC content of 61.4%. The results of the genomic analysis are reported in Table 6. For all genomes, the species already identified through specific *gyrB* sequence analysis was confirmed. The most represented resistance gene across all the analyzed isolates was *blaOXA-427*, providing resistance to beta-lactams, present in 6 out of the 18 isolates, followed by *cphA5* (*n* = 3) and *cphA1* (*n* = 2) genes, encoding subclass B2 metallolactamases, described to exhibit narrow-spectrum activity against carbapenems (Boyd et al., 2020). The *ampH* gene providing resistance to amoxicillin was also found in one isolate (B1738). Besides genes providing resistance to beta-lactamases, *tet(E)* gene was retrieved in three isolates exhibiting resistance to tetracycline (B1154, B1519, and B1722) and *dfrA1* resistance gene was retrieved in one isolate exhibiting resistance to sulfamethoxazole-trimethoprim (B1759). Chromosomal mutations and efflux pump–mediated mechanisms were not investigated in this study; however, these mechanisms could explain the resistance phenotypes observed for nalidixic acid (in isolates B1245, B995, B1550, B1082, B1578, B1759, B1761, and B1766), cefotaxime (in isolates B1004 and B1770), sulfamethoxazole-trimethoprim (in isolates B1248 and B998), colistin (in isolate B1738) and tetracycline (in isolate B1754).

**Table 6:** Genomic analysis of the *Aeromonas* spp. isolates classified as non-wild-type (NWT) based on the calculated MIC values and epidemiological cut-offs (COwt).

ID Sample	Bacterial Species	Seafood category samples	Fish species	Sold form (fresh vs. frozen)	Raising claims (farm-raised vs. wild-caught)	FAO Zone	NAL	CEF	COL	E	FFC	GEN	SXT	TE	Antibiotic resistance genes
B1154	*A. rivipollensis*	C	Squid	Fresh	Wild-caught	FAO 34	WT	WT	WT	WT	WT	WT	WT	NWT	*tet(E)*
B1245	*A. salmonicida*	C	Cuttlefish	Fresh	Wild-caught	FAO 37.2.1	NWT	WT	WT	WT	WT	WT	WT	WT	*cphA5*
B995	*A. rivipollensis*	C	Squid	Fresh	Wild-caught	FAO 27.7	NWT	WT	WT	WT	WT	WT	WT	WT	—
B1550	*A. media*	C	Flying squid	Fresh	Wild-caught	FAO 27.8\u00B0C	NWT	WT	WT	WT	WT	WT	WT	WT	*blaOXA-427*
B1248	*A. rivipollensis*	D	Cod	Fresh	Wild-caught	FAO 27. VIII. C	WT	WT	WT	WT	WT	WT	NWT	WT	—
B1519	*A. media*	D	Cod	Frozen	Wild-caught	Southeast Atlantic Ocean	WT	WT	WT	WT	WT	WT	WT	NWT	*blaOXA-427, tet(E)*
B998	*A. rivipollensis*	D	Cod	Fresh	Wild-caught	FAO 37	WT	WT	WT	WT	WT	WT	NWT	WT	—
B1004	*A. media*	F	Clam	Fresh	Wild-caught	FAO 37	WT	NWT	WT	WT	WT	WT	WT	WT	*blaOXA-427*
B1082	*A. media*	G	Shrimp	Frozen	Wild-caught	FAO 41	NWT	WT	WT	WT	WT	WT	WT	WT	—
B1578	*A. salmonicida*	G	Prawn	Fresh	Wild-caught	FAO 27.3 A	NWT	WT	WT	WT	WT	WT	WT	WT	*cphA5*
B1738	*A. hydrophila*	B	Anchovy	Fresh	Wild-caught	FAO 37.2.1	WT	WT	NWT	WT	WT	WT	WT	WT	*ampH, cphA1*
B1739	*A. salmonicida*	B	Anchovy	Fresh	Wild-caught	FAO 37.2.1	WT	WT	WT	WT	WT	WT	WT	NWT	*cphA5, tet(E)*
B1754	*A. rivipollensis*	E	Salmon	Fresh	Farm-raised	Norway	WT	WT	WT	WT	WT	WT	WT	NWT	—
B1759	*A. bivalvium*	F	Clam	Fresh	Wild-caught	FAO 37.2.1	NWT	WT	WT	WT	WT	WT	NWT	WT	*blaOXA-427, dfrA1*
B1761	*A. media*	F	Clam	Fresh	Wild-caught	FAO 37.2.1	NWT	WT	WT	WT	WT	WT	WT	WT	*blaOXA-427*
B1766	*A. salmonicida*	G	Prawn	Frozen	Wild-caught	FAO 27	NWT	WT	WT	WT	WT	WT	WT	WT	*cphA1*
B1770	*A. media*	D	Cod	Fresh	Wild-caught	FAO 37.2.1	WT	NWT	WT	WT	WT	WT	WT	WT	*blaOXA-427*
B1772	*A. rivipollensis*	F	Mussel	Fresh	Farm-raised	FAO 37.2.1	WT	WT	WT	WT	WT	WT	WT	NWT	*tet(E)*

Nalidixic acid (NAL), cefotaxime (CEF), colistin (COL), erythromycin (E), florfenicol (FFC), gentamicin (GEN), sulfamethoxazole-trimethoprim (SXT), tetracycline (TE).

## Discussion

The use of an indicator bacterium for monitoring antimicrobial resistance represents an excellent method to simplify and standardize data collection. This approach facilitates data comparison, enabling a better understanding and control of the evolution of antimicrobial resistance. While terrestrial veterinary medicine widely employs *Escherichia coli* as an indicator bacterium, no such standard bacterium exists for aquatic environments. Among the potential aquatic indicator bacteria, several studies have proposed the use of *Aeromonas* spp. (Usui et al., 2016; Grilo et al., 2020; Aerts et al., 2024). *Aeromonas* spp. is ubiquitous in aquatic environments, both freshwater and marine (Janda and Abbott, 2010; Baron et al., 2017), and it can be isolated at any time of the year (Gomes et al., 2021). Moreover, *Aeromonas* can acquire antibiotic resistance mechanisms and has the potential for horizontal gene transfer, so it may be a good candidate for tracking the spread of antibiotic resistance in the water (Gomes et al., 2021). *Aeromonas* has been accepted as an indicator organism for monitoring water quality and sewage pollution, and it is suitable for evaluating the prevalence, development, and spread of AMR in the aquatic environment (Grilo et al., 2020). Several researchers have used *Aeromonas* as indicator bacteria for antimicrobial susceptibility in aquatic environments (Usui et al., 2016; Skwor et al., 2020). In this study, more than 60% of samples tested positive for *Aeromonas* spp., confirming their widespread presence in fishery products intended for consumption.

A review published in *Nature* regarding trends in antibiotic resistance in fishery products in Asia over the past two decades revealed that the most frequently detected bacterial genera were *Vibrio* spp. and *Aeromonas* spp., together accounting for nearly 50% of isolates (Schar et al., 2021). Similarly, in the Mediterranean basin, the prominence of Aeromonas spp. has also been documented, with studies by Pepi and Focardi (2021) and Gambino et al. (2022) reporting the widespread occurrence of this genus among antibiotic-resistant bacteria detected in aquaculture and coastal marine environments. This finding strengthens the potential validity of using *Aeromonas* spp. as an indicator bacterium. In the present study, we found a significant difference between the prevalence of *Aeromonas* spp. in fresh and in frozen products. The prevalence of *Aeromonas* spp. in fresh products exceeded 80%, while in frozen products, it was approximately 24%. This difference was also significant within categories sampled both as fresh and frozen products. For example, in category C, a prevalence difference greater than 70% was observed between fresh and frozen samples, according with the literature (Park et al., 2021). These findings underscore a limitation in the use of *Aeromonas* spp. as an indicator bacterium in frozen fishery products. This constraint could potentially be mitigated by enhancing the sensitivity of the cultural analysis, for instance by increasing the sample volume used during the pre-enrichment phase.

When analyzing the data by product category, divided into fresh and frozen, no differences in the prevalence of *Aeromonas* spp. were observed, except for fresh products in category B, where prevalence was slightly lower than in category A. However, it should be noted that the number of samples in category B was lower than planned and not adequately distributed over the calendar year, as fresh anchovies were not available during the winter months. This limitation affected the prevalence data for this category.

The widespread presence of *Aeromonas* spp. in fishery products is also significant due to its zoonotic potential. In this study, 100 isolates selected for MIC testing were identified at the species level via *gyrB* gene sequencing. Most identified species were *A. salmonicida* (47%) and *A. media* (21%). Although less prevalent, species considered primary causes of clinical manifestations in humans, such as *A. caviae* (1%), *A. veronii* (6%), and *A. hydrophila* (2%), were also identified (Fernández-Bravo and Figueras, 2020). The presence of these *Aeromonas* species in retail products confirms that consuming raw fishery products or potential cross-contaminations poses a risk to consumer health.

One objective of this study was to evaluate antibiotic resistance profiles against major antimicrobial classes in a collection of 100 *Aeromonas* spp. isolates from fishery products sold in retail markets. This is the first study of this type conducted using Italian isolates. CLSI provides antimicrobial breakpoints only for *A. salmonicida* (CLSI, 2020a) to two antimicrobials (oxytetracycline and oxolinic acid). So, antibiotic resistance evaluation was based on epidemiological cut-off proposed for this microorganism in the CLSI VET 04 document (CLSI, 2020b). It should be noted that the *Aeromonas* genus is naturally resistant to certain beta-lactams, including ampicillin, amoxicillin+clavulanic acid, and cefazolin (Bakken et al., 1988; CLSI, 2018). This represents an intrinsic limitation to its use as an antimicrobial resistance indicator. Of the eight molecules tested in this study, epidemiological cut-off are available for tetracyclines class, cephalosporin, florfenicol, gentamicin, and sulfamethoxazole-trimethoprim. The protocol reported in the literature (Kronvall, 2010) was used to establish the COwt values for *Aeromonas*. The results obtained in the present study can be used for comparison with those reported in the literature (Baron et al., 2017; Lin et al., 2022) to verify the reproducibility of the method and to determine whether COwt values are influenced by the sold form (fresh or frozen) of the bacterial strains. Additionally, this study can provide breakpoints for determining the epidemiological characteristics of *Aeromonas* AMR to antimicrobials of greatest concern in retail products.

The COwt obtained in this study was compared with the interpretative thresholds reported in CLSI (2020b). The results showed that the COwt value of *Aeromonas* spp. to florfenicol, cefotaxime, tetracycline and gentamicin were consistent to the corresponding result of CLSI, exceedingly one or two drug dilution gradients. Moreover, the COwt values obtained in this study were consistent with those previously reported by Baron et al. (2017). Specifically, the COwt for *Aeromonas* spp. against nalidixic acid matched exactly the value described by Baron et al. (2017), while the COwt for erythromycin closely aligned with the findings of both Baron et al. (2017); Lin et al., (2022), differing by only a single dilution gradient. Larger difference was observed for sulfamethoxazole-trimethoprim: the COwt obtained in this study (4 μg/mL) was higher than the corresponding result of CLSI and the COwt value reported by Baron et al. (2017) (0.25 μg/mL) and Lin et al., (2022) (1 μg/mL).

Epidemiological cut-off values (ECOFFs) were calculated for *Aeromonas* spp. rather than individual species, due to the limited number of isolates per species, as CLSI recommends at least 100 isolates per species for reliable ECOFF calculation. This choice aimed to propose a cost-effective yet reliable epidemiological cut-off. According to the clinical breakpoints, no clinical resistance was detected among the 100 isolates for cefotaxime or gentamicin, with all isolates below the respective thresholds of 4 μg/mL and 16 μg/mL. For tetracycline, four resistant isolates were detected, all identified as *A. media*, with three isolates showing MIC values of 16 μg/mL and one isolate showing an MIC of 32 μg/mL. For sulfamethoxazole-trimethoprim, three resistant isolates were identified, two of which were *A. media* with MIC values of 32 μg/mL and 128 μg/mL, and one *A. bivalvium* isolate with an MIC of 256 μg/mL.

These results suggest that the reduced susceptibility observed for tetracyclines, sulfonamides, and quinolones may stem from prolonged or repeated exposure to these antibiotic classes in aquaculture environments. The persistence of antibiotic residues in water and sediments can create selective pressures that favor the survival and proliferation of resistant strains. Such environmental conditions may also facilitate horizontal gene transfer among microbial populations, thereby enhancing the spread of resistance determinants within aquaculture systems.

Genomic analyses of isolates classified as NWT for at least one of the tested antimicrobial compounds confirmed the species classification. As expected, the analysis of acquired AMR genes allowed to identify a wide distribution of genes encoding beta-lactamases, mainly *blaOXA-427* and *cphA* genes, as well as *ampH* gene in one isolate. Interestingly, also *tet(E)* and *dfrA1* genes were retrieved in isolates which exhibited phenotypic resistance to tetracycline and sulfamethoxazole-trimethoprim, respectively. Such AMR genes could have been acquired through horizontal gene transfer and might be transferred to other microorganisms by mobile genetic elements, potentially contributing to AMR spread. On the other hand, the identified resistance to nalidixic acid in eight isolates, to cefotaxime in two isolates as well as to colistin and tetracycline in one isolate each, could not be explained with the presence of acquired AMR genes. Indeed, resistance to these compounds may arise through the accumulation of chromosomal mutations in genes encoding target proteins, as documented for nalidixic acid, or through mechanisms such as efflux pump activation or reduced membrane permeability—processes that can be triggered by exposure to antimicrobial agents, including those used in aquaculture settings. Further investigations would be required to elucidate the specific resistance mechanisms operating in these isolates.

In conclusion, although the natural resistance to penicillins, the difficulty in identifying the species and finally, the greater difficulty in isolation from frozen matrices emerged in our study, can represent limitations in the use of this bacterial genus as an indicator, *Aeromonas* spp. shows potential as an indicator bacterium for monitoring antimicrobial resistance in fishery products.

Future research should focus on expanding available clinical breakpoints and studying resistance mechanisms in greater detail to optimize monitoring strategies in aquatic environments.

## Conclusion

*Aeromonas* spp. was proposed as a potential indicator of antimicrobial susceptibility for aquatic environment by several authors (Usui et al., 2016; Varela et al., 2016). Its abundance in aquatic environments and its ability to acquire new genetic material through conjugation processes (Bello-López et al., 2019) makes it a “sentinel” microorganism to monitor the development and spread of antibiotic resistance in the aquatic environment. Moreover, the high presence of *Aeromonas* spp. in the various fish categories evaluated in this study strengthens its candidacy as an indicator bacterium for antibiotic resistance.

In this study, although most of the *Aeromonas* spp. isolates tested did not show resistance to antibiotics, the presence of resistant isolates for some of the tested molecules was detected. The finding of these on fish products for consumption needs particular attention, since it could represent a risk for Public Health. Moreover, the presence in fish of isolates resistant to antibiotic classes most commonly used in aquaculture underlines the importance of surveillance of antibiotic resistance and consumption of antimicrobials in this sector.

In order to make *Aeromonas* spp. an effective indicator of resistance in the aquatic environment, it will be necessary to improve the harmonization of methods for detecting, identifying and evaluating resistance at a global level. The establishment of reliable epidemiological cut-offs will allow a better understanding of the spread of the problem of antibiotic resistance in aquatic environments and in fish products. The use of new methods, such as Whole Genome Sequencing, the study of mobile genetic elements and the analysis of the environmental resistome, can give a further boost to the study of the development of resistance.

## Data Availability

The raw sequencing data has been submitted to EMBL-ENA (project Accession numbers: PRJEB96839).

## Footnotes

1
www.eucast.org

2
https://epitools.ausvet.com.au/

3
https://aries.iss.it/platform/

4
https://pubmlst.org/species-id

## Supplementary material

SUPPLEMENTARY FIGURE S1 Distribution of MIC values among *Aeromonas* spp. isolates for each antibiotic molecule. The bar graphs show the percentage of *Aeromonas* spp. isolates for each MIC value.
