# Supplementary figures and images for: Aeromonas spp. as possible bacterial indicator for monitoring antibiotic resistance in seafood
Source: Front Microbiol. 2026 Jan 20;16:1721645. doi: 10.3389/fmicb.2025.1721645 (PMC12864392; doi:10.3389/fmicb.2025.1721645)

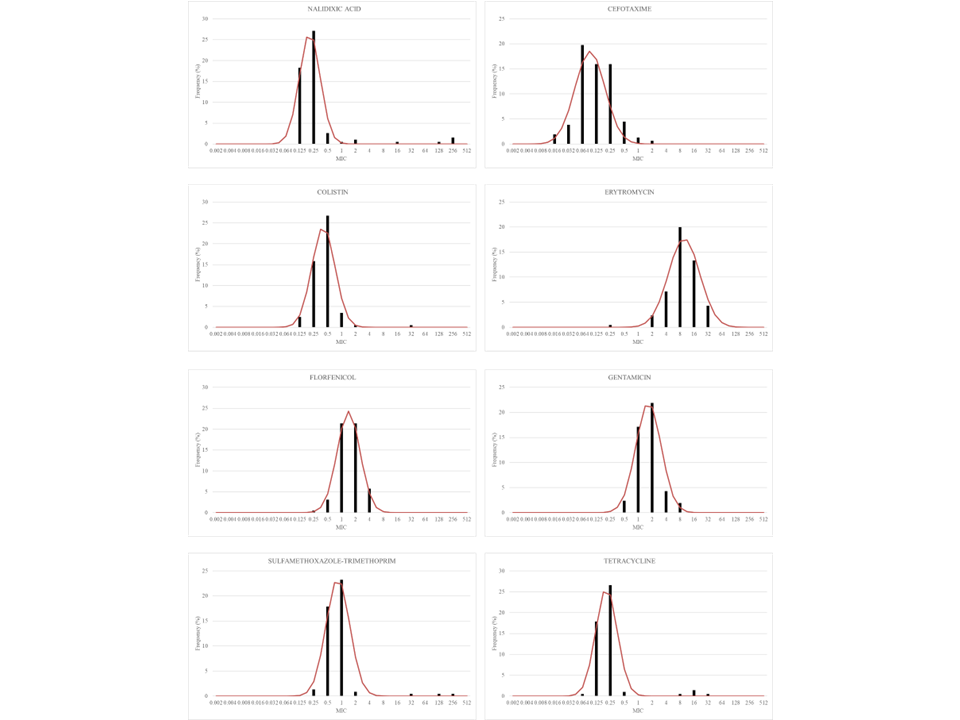

Supplement: SUPPLEMENTARY FIGURE S1 — Distribution of MIC values among Aeromonas spp. isolates for each antibiotic molecule. The bar graphs show the percentage of Aeromonas spp. isolates for each MIC value. [file Image_1.TIF]
