# Supplementary material for: Aeromonas spp. as possible bacterial indicator for monitoring antibiotic resistance in seafood
Source: Front Microbiol. 2026 Jan 20;16:1721645. doi: 10.3389/fmicb.2025.1721645 (PMC12864392; doi:10.3389/fmicb.2025.1721645)
Supplement: Supplementary file 2 [file Table_1.docx]

**Table S1**: Information relating to each of the 100 *Aeromonas* spp. isolates selected for the study.

| **ID Sample** | **Bacteria species** | **Seafood**  **category samples** | **Fish species** | **Sold form**  **(fresh vs. frozen)** | **Raising claims**  **(farm-raised vs. wild-caught)** | **FAO Zone** | **Sampling period** |
| --- | --- | --- | --- | --- | --- | --- | --- |
| B1042 | *Aeromonas salmonicida* | A | Sea bass | Fresh | Farm-raised | Italy | July 2020 |
| B1091 | *Aeromonas salmonicida* | A | Sea bass | Fresh | Farm-raised | Greece | August 2020 |
| B1222 | *Aeromonas hydrophila* | A | Sea bass | Fresh | Farm-raised | Italy | October 2020 |
| B1213 | *Aeromonas salmonicida* | A | Sea bream | Fresh | Wild-caught | FAO 37.2.1 | October 2020 |
| B1217 | *Aeromonas crassostreae* | A | Sea bream | Fresh | Farm-raised | Greece | October 2020 |
| B1487 | *Aeromonas salmonicida* | A | Sea bream | Fresh | Farm-raised | Italy | February 2021 |
| B1537 | *Aeromonas media* | A | Sea bream | Fresh | Wild-caught | FAO 37.2.2 | March 2021 |
| B1595 | *Aeromonas allosaccharophila* | A | Sea bream | Fresh | Farm-raised | Malta | April 2021 |
| B1543 | *Aeromonas salmonicida* | A | Sea bass | Fresh | Farm-raised | Italy | March 2021 |
| B991 | *Aeromonas media* | B | Clam | Fresh | Farm-raised | Italy | June 2020 |
| B1039 | *Aeromonas salmonicida* | B | Anchovy | Fresh | Wild-caught | FAO 37 | July 2020 |
| B1363 | *Aeromonas media* | B | Anchovy | Fresh | Wild-caught | FAO 37.2 | December 2020 |
| B1546 | *Aeromonas salmonicida* | B | Anchovy | Fresh | Wild-caught | FAO 37.1.3 | March 2021 |
| B1154 | *Aeromonas rivipollensis* | C | Squid | Fresh | Wild-caught | FAO 34 | September 2020 |
| B1233 | *Aeromonas salmonicida* | C | Octopus | Fresh | Wild-caught | FAO 37.1.1 | October 2020 |
| B997 | *Aeromonas media* | C | Cuttlefish | Fresh | Wild-caught | FAO 27 | June 2020 |
| B1245 | *Aeromonas salmonicida* | C | Cuttlefish | Fresh | Wild-caught | FAO 37.2.1 | October 2020 |
| B1056 | *Aeromonas salmonicida* | C | Cuttlefish | Fresh | Wild-caught | FAO 37.2 | July 2020 |
| B995 | *Aeromonas rivipollensis* | C | Squid | Fresh | Wild-caught | FAO 27.7 | June 2020 |
| B1241 | *Aeromonas salmonicida* | C | Flying squid | Fresh | Wild-caught | FAO 27.IX | October 2020 |
| B1550 | *Aeromonas media* | C | Flying squid | Fresh | Wild-caught | FAO 27.8 C | March 2021 |
| B1432 | *Aeromonas salmonicida* | D | Cod | Fresh | Wild-caught | FAO 37.2.1 | January 2021 |
| B1440 | *Aeromonas salmonicida* | D | Cod | Fresh | Wild-caught | FAO 27.7 | January 2021 |
| B1495 | *Aeromonas media* | D | Cod | Fresh | Wild-caught | FAO 27.VII.A | February 2021 |
| B1248 | *Aeromonas rivipollensis* | D | Cod | Fresh | Wild-caught | FAO 27.VIII.C | October 2020 |
| B1197 | *Aeromonas veronii* | D | Cod | Frozen | Wild-caught | FAO 47 | September 2020 |
| B1519 | *Aeromonas media* | D | Cod | Frozen | Wild-caught | Southeast Atlantic Ocean | February 2021 |
| B1586 | *Aeromonas veronii* | D | Cod | Frozen | Wild-caught | Atlantic Ocean | March 2021 |
| B998 | *Aeromonas rivipollensis* | D | Cod | Fresh | Wild-caught | FAO 37 | June 2020 |
| B1340 | *Aeromonas salmonicida* | D | Cod | Frozen | Wild-caught | FAO 47 | November 2020 |
| B1515 | *Aeromonas salmonicida* | D | Cod | Frozen | Wild-caught | Pacific Ocean | February 2021 |
| B999 | *Aeromonas media* | E | Salmon | Fresh | Wild-caught | FAO 27 | June 2020 |
| B1067 | *Aeromonas salmonicida* | E | Salmon | Fresh | Farm-raised | Norway | July 2020 |
| B1110 | *Aeromonas salmonicida* | E | Salmon | Fresh | Farm-raised | Norway | August 2020 |
| B1446 | *Aeromonas salmonicida* | E | Salmon | Fresh | Farm-raised | Norway | January 2021 |
| B1497 | *Aeromonas salmonicida* | E | Salmon | Fresh | Farm-raised | Norway | February 2021 |
| B1561 | *Aeromonas salmonicida* | E | Salmon | Fresh | Farm-raised | Norway | March 2021 |
| B1266 | *Aeromonas salmonicida* | E | Salmon | Frozen | Wild-caught | FAO 61 | October 2020 |
| B1521 | *Aeromonas salmonicida* | E | Salmon | Frozen | Farm-raised | Denmark | February 2021 |
| B1630 | *Aeromonas salmonicida* | E | Salmon | Frozen | Wild-caught | Atlantic Ocean | April 2021 |
| B1004 | *Aeromonas media* | F | Clam | Fresh | Wild-caught | FAO 37 | June 2020 |
| B1072 | *Aeromonas veronii* | F | Clam | Fresh | Farm-raised | Italy | July 2020 |
| B1185 | *Aeromonas veronii* | F | Clam | Fresh | Wild-caught | FAO 37 | September 2020 |
| B1452 | *Aeromonas bivalvium* | F | Clam | Fresh | Wild-caught | Mare Adriatico | January 2021 |
| B1570 | *Aeromonas media* | F | Clam | Fresh | Farm-raised | Italy | March 2021 |
| B1008 | *Aeromonas bivalvium* | G | Shrimp | Frozen | Wild-caught | FAO 27 | June 2020 |
| B1404 | *Aeromonas salmonicida* | G | Shrimp | Frozen | Wild-caught | Indian Ocean | December 2020 |
| B1082 | *Aeromonas media* | G | Shrimp | Frozen | Wild-caught | FAO 41 | July 2020 |
| B1632 | *Aeromonas rivipollensis* | G | Shrimp | Frozen | Farm-raised | Italy | April 2021 |
| B1010 | *Aeromonas salmonicida* | G | Prawn | Frozen | Wild-caught | FAO 87 | June 2020 |
| B1511 | *Aeromonas salmonicida* | G | Langoustine | Fresh | Wild-caught | FAO 27.II.A | February 2021 |
| B1626 | *Aeromonas molluscorum* | G | Langoustine | Fresh | Wild-caught | FAO 41 | April 2021 |
| B1578 | *Aeromonas salmonicida* | G | Langoustine | Fresh | Wild-caught | FAO 27.3 A | March 2021 |
| B1585 | *Aeromonas molluscorum* | G | Langoustine | Frozen | Wild-caught | FAO 27 | March 2021 |
| B1731 | *Aeromonas media* | A | Sea bass | Fresh | Farm-raised | Italy | June 2020 |
| B1732 | *Aeromonas salmonicida* | A | Sea bass | Fresh | Farm-raised | Italy | February 2021 |
| B1733 | *Aeromonas bivalvium* | A | Sea bass | Fresh | Farm-raised | Albania | December 2020 |
| B1734 | *Aeromonas bivalvium* | A | Sea bass | Fresh | Farm-raised | Greece | August 2020 |
| B1735 | *Aeromonas salmonicida* | B | Anchovy | Fresh | Wild-caught | FAO 37.2.1 | January 2021 |
| B1736 | *Aeromonas salmonicida* | B | Anchovy | Fresh | Wild-caught | FAO 37.2.1 | January 2021 |
| B1737 | *Aeromonas veronii* | B | Anchovy | Fresh | Wild-caught | FAO 37.2.1 | March 2021 |
| B1738 | *Aeromonas hydrophila* | B | Anchovy | Fresh | Wild-caught | FAO 37.2.1 | July 2020 |
| B1739 | *Aeromonas salmonicida* | B | Anchovy | Fresh | Wild-caught | FAO 37.2.1 | December 2020 |
| B1740 | *Aeromonas media* | B | Anchovy | Fresh | Wild-caught | FAO 37.2.1 | April 2021 |
| B1741 | *Aeromonas molluscorum* | B | Anchovy | Fresh | Wild-caught | FAO 37.2.1 | April 2021 |
| B1742 | *Aeromonas salmonicida* | B | Anchovy | Fresh | Wild-caught | FAO 37.2.1 | April 2021 |
| B1743 | *Aeromonas salmonicida* | B | Anchovy | Fresh | Wild-caught | FAO 37.2.1 | April 2021 |
| B1744 | *Aeromonas media* | C | Cuttlefish | Fresh | Wild-caught | FAO 27 VII | December 2020 |
| B1745 | *Aeromonas salmonicida* | C | Flying squid | Fresh | Wild-caught | FAO 37.4 | June 2020 |
| B1746 | *Aeromonas salmonicida* | C | Flying squid | Fresh | Wild-caught | FAO 37.2.1 | August 2020 |
| B1747 | *Aeromonas bivalvium* | C | Flying squid | Fresh | Wild-caught | FAO 37.4 | December 2020 |
| B1748 | *Aeromonas salmonicida* | C | Flying squid | Fresh | Wild-caught | FAO 87 | January 2021 |
| B1749 | *Aeromonas caviae* | D | Cod | Frozen | Wild-caught | FAO 61 | October 2020 |
| B1750 | *Aeromonas media* | D | Cod | Fresh | Wild-caught | FAO 37.2.1 | April 2021 |
| B1751 | *Aeromonas salmonicida* | D | Cod | Fresh | Wild-caught | FAO 37.2.1 | October 2020 |
| B1752 | *Aeromonas molluscorum* | E | Salmon | Fresh | Farm-raised | Norway | June 2020 |
| B1753 | *Aeromonas salmonicida* | E | Salmon | Fresh | Farm-raised | Norway | August 2020 |
| B1754 | *Aeromonas rivipollensis* | E | Salmon | Fresh | Farm-raised | Norway | September 2020 |
| B1755 | *Aeromonas media* | E | Salmon | Fresh | Farm-raised | Iceland | December 2020 |
| B1756 | *Aeromonas crassostreae* | F | Mussel | Fresh | Farm-raised | FAO 37.2.1 | June 2020 |
| B1757 | *Aeromonas media* | F | Mussel | Fresh | Farm-raised | Spain | December 2020 |
| B1758 | *Aeromonas crassostreae* | F | Clam | Fresh | Wild-caught | FAO 37.2.1 | July 2020 |
| B1759 | *Aeromonas bivalvium* | F | Clam | Fresh | Wild-caught | FAO 37.2.1 | September 2020 |
| B1760 | *Aeromonas salmonicida* | F | Clam | Fresh | Wild-caught | FAO 37.2.1 | December 2020 |
| B1761 | *Aeromonas media* | F | Clam | Fresh | Wild-caught | FAO 37.2.1 | January 2021 |
| B1762 | *Aeromonas allosaccharophila* | F | Clam | Fresh | Wild-caught | FAO 37.2.1 | April 2021 |
| B1763 | *Aeromonas bivalvium* | F | Clam | Fresh | Farm-raised | FAO 37 | February 2021 |
| B1764 | *Aeromonas salmonicida* | G | Shrimp | Fresh | Wild-caught | FAO 41 | July 2020 |
| B1765 | *Aeromonas salmonicida* | G | Prawn | Frozen | Farm-raised | Ecuador | August 2020 |
| B1766 | *Aeromonas salmonicida* | G | Langoustine | Frozen | Wild-caught | FAO 27 | February 2021 |
| B1767 | *Aeromonas veronii* | G | Langoustine | Frozen | Wild-caught | FAO 27 | September 2020 |
| B1768 | *Aeromonas salmonicida* | A | Sea bass | Fresh | Farm-raised | Italy | May 2021 |
| B1769 | *Aeromonas salmonicida* | B | Anchovy | Fresh | Wild-caught | FAO 37.2.1 | May 2021 |
| B1770 | *Aeromonas media* | D | Cod | Fresh | Wild-caught | FAO 37.2.1 | May 2021 |
| B1771 | *Aeromonas salmonicida* | E | Salmon | Fresh | Farm-raised | Norway | May 2021 |
| B1772 | *Aeromonas rivipollensis* | F | Mussel | Fresh | Farm-raised | FAO 37.2.1 | May 2021 |
| B1637 | *Aeromonas media* | A | Sea bass | Fresh | Farm-raised | Greece | May 2021 |
| B1659 | *Aeromonas salmonicida* | B | Anchovy | Fresh | Wild-caught | FAO 37.1.2 | May 2021 |
| B1697 | *Aeromonas salmonicida* | B | Anchovy | Fresh | Wild-caught | FAO 37.1.3 | June 2021 |
| B1645 | *Aeromonas media* | E | Salmon | Fresh | Farm-raised | Norway | May 2021 |
