# Supplementary material for: Aeromonas spp. as possible bacterial indicator for monitoring antibiotic resistance in seafood
Source: Front Microbiol. 2026 Jan 20;16:1721645. doi: 10.3389/fmicb.2025.1721645 (PMC12864392; doi:10.3389/fmicb.2025.1721645)
Supplement: Supplementary file 3 [file Table_2.docx]

**Table S2**. Number and distribution of *Aeromonas* spp. isolates from seafood samples.

| **Seafood**  **category**  **samples** | ***A. allosaccharophila*** | ***A. bivalvium*** | ***A. caviae*** | ***A. crassostreae*** | ***A. hydrophila*** | ***A. media*** | ***A. molluscorum*** | ***A. rivipollensis*** | ***A. salmonicida*** | ***A. veronii*** | **Total** |
| --- | --- | --- | --- | --- | --- | --- | --- | --- | --- | --- | --- |
| **A** | **1** | **2** |  | **1** | **1** | **3** |  |  | **7** |  | **15** |
| Sea bass |  | 2 |  |  | 1 | 2 |  |  | 5 |  | 10 |
| Sea bream | 1 |  |  | 1 |  | 1 |  |  | 2 |  | 5 |
| **B** |  |  |  |  | **1** | **3** | **1** |  | **10** | **1** | **16** |
| Anchovy |  |  |  |  | 1 | 2 | 1 |  | 10 | 1 | 15 |
| Clam |  |  |  |  |  | 1 |  |  |  |  | 1 |
| **C** |  | **1** |  |  |  | **3** |  | **2** | **7** |  | **13** |
| Cuttlefish |  |  |  |  |  | 2 |  |  | 2 |  | 4 |
| Flying squid |  | 1 |  |  |  | 1 |  |  | 4 |  | 6 |
| Octopus |  |  |  |  |  |  |  |  | 1 |  | 1 |
| Squid |  |  |  |  |  |  |  | 2 |  |  | 2 |
| **D** |  |  | **1** |  |  | **4** |  | **2** | **5** | **2** | **14** |
| Cod |  |  | 1 |  |  | 4 |  | 2 | 5 | 2 | 14 |
| **E** |  |  |  |  |  | **3** | **1** | **1** | **10** |  | **15** |
| Salmon |  |  |  |  |  | 3 | 1 | 1 | 10 |  | 15 |
| **F** | **1** | **3** |  | **2** |  | **4** |  | **1** | **1** | **2** | **14** |
| Clam | 1 | 3 |  | 1 |  | 3 |  |  | 1 | 2 | 11 |
| Mussel |  |  |  | 1 |  | 1 |  | 1 |  |  | 3 |
| **G** |  | **1** |  |  |  | **1** | **2** | **1** | **7** | **1** | **13** |
| Prawn |  |  |  |  |  |  |  |  | 2 |  | 2 |
| Langoustine |  |  |  |  |  |  | 2 |  | 3 | 1 | 6 |
| Shrimp |  | 1 |  |  |  | 1 |  | 1 | 2 |  | 5 |
| **Total** | **2** | **7** | **1** | **3** | **2** | **21** | **4** | **7** | **47** | **6** | **100** |
